# Impact of ankylosing spondylitis on sexual function: A systematic review and meta-analysis

**DOI:** 10.3892/etm.2015.2239

**Published:** 2015-01-29

**Authors:** YA-FEI LIU, HUI DONG, ZHE CHEN, YU WANG, SHENG-HAO TU

**Affiliations:** Department of Integrated Traditional Chinese and Western Medicine, Tongji Hospital, Tongji Medical College, Huazhong University of Science and Technology, Wuhan, Hubei 430030, P.R. China

**Keywords:** ankylosing spondylitis, sexual function, systematic review, meta-analysis

## Abstract

A number of studies have reported the association of sexual problems with ankylosing spondylitis (AS); however, the results have been conflicting. The present study aimed to investigate the impact of AS on sexual function. To develop a more comprehensive understanding of sexual function in patients with AS, a systematic review and meta-analysis of the literature up to 2013 was conducted. Studies that assessed the impact of AS on sexual function by adopting the International Index of Erectile Function or the Female Sexual Function Index (FSFI) scoring system were included. Statistical analysis was performed using Review Manager statistical software (version 5.2). The weighted mean differences were calculated by employing a fixed or random effects model. A total of 484 cases from five studies were identified as being well-documented and included in the meta-analysis. Compared with healthy controls, male patients with AS have a significant reduction in sexual function scores of erectile function (−3.07), orgasmic function (−1.17), sexual drive (−0.72) and intercourse satisfaction (−1.89). Female patients with AS have a lower FSFI score in domains of desire (−0.34) and arousal (−0.87). In conclusion, AS has a certain impact on the sexual function of male patients. AS appears to have a greater influence on the sexual function of males compared with that of females. However, the mechanism by which AS affects sexual function requires further evaluation by further studies of a larger population of patients.

## Introduction

Ankylosing spondylitis (AS) is a systemic chronic rheumatic disease that presents with inflammatory back pain, asymmetrical peripheral arthritis, enthesitis and extra-articular features (1). Apart from spinal stiffness and loss of spinal mobility, AS has a considerable impact on the quality of life (QoL) of patients. Doctors often concentrate on skeletal damage and measures of pain, whereas most AS patients focus on feeling well and the capability to live a full life (2). AS affects patients’ health-related QoL, work life, relationships with family and spouse and expectations (3). Sexuality has been described as an essential part of the whole person, and sexual expression has been cited as a crucial part of an individual’s self identity (4). Sexual function plays a significant role in QoL and it is particularly important for patients with AS as it mainly affects young individuals.

The effect of rheumatologic diseases, particularly AS, on sexual function has been explored (5,6). A previous study demonstrated that patients with erectile dysfunction (ED) were at a higher risk for AS (odds ratio=2.19) (7). A population-based study demonstrated that ED was associated with AS (8). However, another study indicated that the prevalence of ED among AS patients was similar to that of normal healthy controls (9). Therefore, whether AS patients harbor an increased risk of sexual dysfunction when compared to normal population remains controversial.

The aim of this study was to summarize the existing evidence and explore the effect of AS on sexual function by conducting a systematic review and meta-analysis.

## Materials and methods

The meta-analysis was reported in accordance with the recommendations of the Preferred Reporting Items for Systemic Review and Meta-Analyses (PRISMA) and the Meta-analysis of Observational Studies in Epidemiology (MOOSE) as closely as possible (10,11).

### Literature search strategy

The following digital databases were searched to identify relevant trials: PubMed, Embase and the Cochrane Library. In addition, Chinese databases were searched, including the CNKI Scholar, VIP, Chinese BioMedicine (CBM) and WanFang databases. Up-to-date information regarding AS-related sexual function up to May 2013 was retrieved from these databases.

Different search strategies were combined, as follows. For the English-language databases, free text such as ‘sexual activity’, ‘sexual function’, ‘impotence’ or ‘erectile dysfunction’ and ‘Marie-Struempell disease’ ‘Bechterew disease’ ‘ankylosing spondylarthritis’ or ‘ankylosing spondylitis’ were used. For the Chinese databases, free text terms were used, including the Chinese translations of terms meaning sexual function, erectile dysfunction and AS. In order to collect an adequate number of trials, the reference lists of relevant publications were also searched to identify additional studies.

### Inclusion and exclusion criteria

The focus of the analysis was on studies of sexual function in AS regardless of gender, publication status or language. Studies were selected for analysis if they satisfied the following criteria: i) Studies that assessed the association between sexual function and AS; ii) the subjects enrolled were diagnosed with AS according to modified New York criteria for AS (12); and iii) sexual function was evaluated using the International Index of Erectile Function (IIEF) (13) or the Female Sexual Function Index (FSFI) (14) scoring system. Studies were eliminated if sexual function was evaluated by other scoring systems.

For repeated studies of the same data, authors of reports were contacted to clarify ambiguity. If the author could not be reached, the first published study was considered as original. Two reviewers selected articles independently. Based on the PRISMA requirements, a flow diagram of the study selection was generated.

### Data extraction

The relevant data was extracted by two independent investigators, and discrepancies between the two abstractors were resolved by consensus, or re-evaluated by a third reviewer. The validated Newcastle-Ottawa Scale instrument was adopted to independently assess the quality of each study (15). A star system was applied to evaluate nonrandomized studies regarding three criteria: Patient selection (0–4 stars), comparability of study groups (0–2 stars) and exposure or outcome assessment (0–3 stars). Studies that achieved a rating of at least six stars were considered to be of the highest quality (16).

The IIEF index covers five domains: Erectile function (EF; six questions with a maximum score of 30), intercourse satisfaction (IS; three questions with a maximum score of 15), orgasmic function (OF), sexual drive (SD) and overall satisfaction (OS) (two questions each with a maximum score of 10). The FSFI index includes domains of sexual desire, arousal, lubrication, orgasm, satisfaction and pain during sexual intercourse. In case of vagueness or absence of outcomes in the articles, the authors were contacted and related data was extracted by consensus if the authors were unavailable.

### Statistical analysis

The correlation of sexual function with AS was evaluated using Review Manager meta-analysis software (version 5.2; Cochrane Collaboration, Copenhagen, Denmark). Weighted mean differences (MDs) and the 95% confidence interval (CI) were calculated for continuous data. A fixed-effect model was applied to combine these MDs to get an overall MD, also known as an effect estimate. A random-effects model was used if heterogeneity across studies was present. Heterogeneity was evaluated via the χ^2^, τ^2^ and Higgins I^2^ tests, and a P level <0.1 was considered significant. A Z score was adopted to assess the overall effect with significance set at P<0.05. Publication bias was evaluated by the Egger test and represented graphically by funnel plots when the number of included trials was ≥5. To minimize the clinical heterogeneity, subgroup analyses for male sexual function and female sexual function were conducted.

## Results

### Study selection

**Figure 1:**
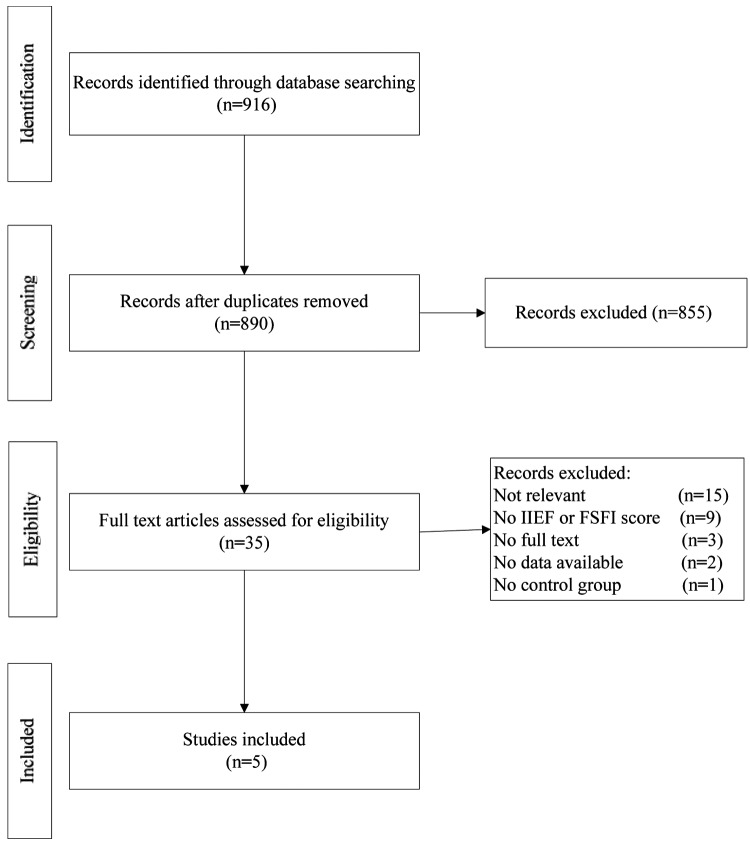
Process of searching for and screening studies. IIEF, International Index of Erectile Function; FSFI, Female Sexual Function Index.

A flowchart of the study selection process is shown in Fig. 1. One study in which the data were presented as the median (minimum-maximum) was excluded (17). According to the selection criteria defined in Materials and methods, the meta-analysis finally included five articles, involving a total of 484 participants of which 232 were patients with AS.

### Study characteristics

Three studies assessed the impact of AS on male sexual function, involving a total of 364 participants of which 172 were male patients with AS (9,18,19). Two studies evaluated sexual function in female patients with AS, involving a total of 120 participants of which 60 were female patients with AS (20,21). The clinical and demographic characteristics of the patients with AS and healthy controls are summarized in Table I. The included studies were published as full text between 2004 and 2013. All studies originated from Turkey and were published in English. The five included studies were of moderate to high quality.

### Publication bias

As the number of included studies in each subgroup was <5, funnel plot analysis and Egger tests were not conducted to test publication bias.

### Male sexual function in AS patients and controls

**Figure 2:**
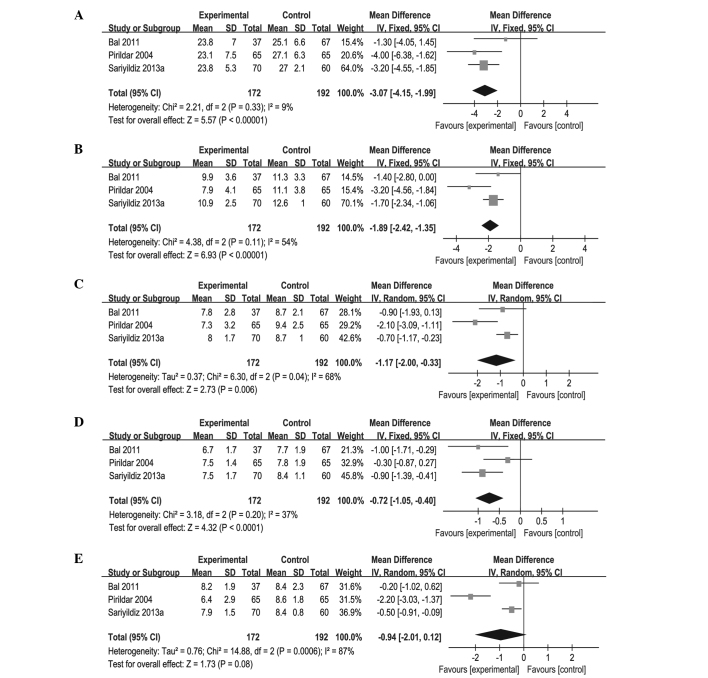
Male sexual function in AS patients and controls. Forest plots of the International Index of Erectile Function. (A) Erectile function, (B) intercourse satisfaction, (C) orgasmic function, (D) sexual drive and (E) overall satisfaction.

The number of trial participants with AS ranged from 37 to 70. The statistical heterogeneity among the studies was found to be significant regarding the results for OF and OS (both P<0.1). Consequently, the random-effects model was applied to pool the results. The pooled results displayed statistical significance with the exception of OS (P=0.08; MD=−0.94; 95% CI: −2.01 to 0.12). Significant differences were identified in scores of EF (P<0.00001; MD=−3.07; 95% CI: −4.15 to −1.99), OF (P=0.006; MD=−1.17; 95% CI: −2.00 to −0.33), SD (P<0.0001; MD=−0.72; 95% CI: −1.05 to −0.40) and IS (P<0.00001; MD=−1.89; 95% CI: −2.42 to −1.35; Fig. 2; Table II).

### Female sexual function in AS patients and controls

**Figure 3:**
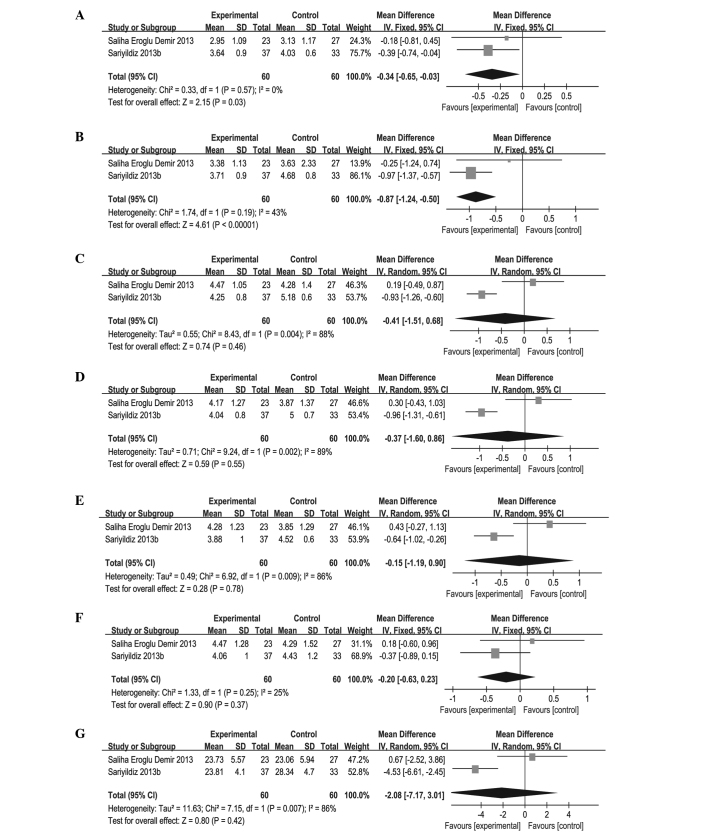
Female sexual function in AS patients and controls. Forest plots of the Female Sexual Function Index (FSFI). (A) Desire, (B) arousal, (C) lubrication, (D) orgasm, (E) satisfaction, (F) pain and (G) total FSFI. AS, ankylosing spondylitis.

The number of trial participants with AS ranged from 23 to 37. There was significant heterogeneity in terms of the domains for total FSFI, lubrication, orgasm and satisfaction (all P<0.1). Accordingly, the random-effects model was applied to combine these domains. The pooled results displayed no significant difference between patients with AS and healthy controls in sexual function, with the exception of desire (P=0.03; MD= −0.34; 95% CI: −0.65 to −0.03) and arousal (P<0.00001; MD= −0.87; 95% CI: −1.24 to −0.50). However, no effects were found on total FSFI (P=0.42; MD= −2.08; 95% CI: −7.17 to 3.01), lubrication (P=0.46; MD= −0.41; 95% CI: −1.51 to 0.68), orgasm (P=0.55; MD= −0.37; 95% CI: −1.60 to 0.86), satisfaction (P=0.78; MD= −0.15; 95% CI: −1.19 to 0.90) and pain (P=0.37; MD= −0.20; 95% CI: −0.63 to 0.23; Fig. 3; Table III).

## Discussion

Increasing evidence indicates that sexual function as an essential component of QoL is influenced by AS. To the best of our knowledge, the present meta-analysis is the first quantitative review analyzing the effect of AS on sexual function. In the meta-analysis, it was found that AS had a certain impact on the sexual function of patients, particularly that of male patients. Five studies with a total of 484 Turkish participants were included. In comparison with healthy controls, the male patients with AS had a lower IIEF score in the domains of EF, OF, SD and IS. The results demonstrated that male patients with AS had a significant reduction in sexual function scores of EF (−3.07), OF (−1.17), SD (−0.72) and IS (−1.89). Compared with healthy controls, female patients with AS have a lower FSFI score in domains of desire (−0.34) and arousal (−0.87).

AS may have a greater impact on male sexual function than on that of females. The possible reasons are as follow. Firstly, it has been reported that males are more often affected by AS than females (22,23) and pertinent studies concerning female sexual function are few. Secondly, female patients had a shorter time of disease duration, older age of disease onset and lower baseline C-reactive protein level (24). Among patients with longstanding AS, there are differences in the clinical manifestations of AS between the genders: Males have significantly more severe radiographic changes and females may have more peripheral arthritis (25). The probability of enjoying sexual activity is diminished in female patients with AS (26). Thirdly, sexual problems tend to be more associated with physical health and aging among males than females (27). In addition to these reasons, neurotransmitters, psychosocial and interpersonal factors (28), and the complexity of sexual dysfunction in females (29) also contribute to the differences in sexual response between males and females.

The pathogenesis of sexual problems in patients with AS is complicated and multifactorial. Several underlying mechanisms might be involved in the effect of AS on sexual function. Firstly, tumor necrosis factor α (TNF-α) plays a pivotal role in the pathogenesis of AS, and TNF-α concentrations are increased in the circulation (30) and synovial tissue (31) in patients with AS. It has been shown that TNF-α acts as a potential candidate in the pathophysiology of ED (32). In addition, a pilot study demonstrated that anti-TNF-α therapy may improve sexual dysfunction in male AS patients (33). Secondly, sexual problems in AS patients might be associated with joint involvement, increased disease activity, decreased functionality, declined health quality and depression (34,35). Poor function, depression, greater disease activity, unemployment and poor self-efficacy have been found to be independently associated with a greater impact on the sexual relationships of patients with AS (36). Bath AS mobility index, impaired social functioning and Bath AS functionality index have been identified to be the most significant causes of impaired sexual function in Chinese AS patients (37). Finally, the toxicity of disease-modifying antirheumatic drugs could contribute to sexual problems. Case reports have shown that methotrexate is associated with reduced libido, ED or impotence (38–40).

Several limitations of the present study should be noted. Firstly, although the number of participants included in this meta-analysis is larger than that in an individual study, the sample size may be not large enough. In the current meta-analysis, numerous studies were excluded due to inconformity with the IIEF or FSFI scoring system. Secondly, all the participants were recruited from Turkish populations. The association of sexual problems and AS may differ in populations from other ethnic origins. Additionally, there was a considerable heterogeneity in the subgroup of female sexual function in AS. It is hypothesized that differences in the quality of studies, participant characteristics and disease activity are responsible for the heterogeneity. In view of this, all of the conclusions require careful consideration.

In conclusion, there is an association between sexual problems and AS. AS appears to have a greater influence on the sexual function of males than that of females. Early diagnosis of sexual dysfunction is essential for AS patients with sexual problems. Therefore, physicians should be aware of the effect of AS on sexual health and it is recommended that special attention is given to all domains of life, not only disease activity and physical function.

## Figures and Tables

**Table I:** Clinical and demographic characteristics of the patients with AS and healthy controls.

Author (ref.)	Number of participants		Age (years)		Characteristics of patients with AS						
		
	Experimental	Control	Experimental	Control	BASFI	BASDAI	DD (years)	DMS (min)	ESR (mm/h)	CRP (mg/dl)	ASQoL
Pirildar (18)	65	65	36.0±8.1	37.0±5.2	5.29±2.56	Not reported	12.2±6.4	220±122	54±23	21±15	Not reported
Bal (9)	37	67	42.8±10.8	43.6±5.9	3.8±2.9	3.92^a^	10.0±9.0	4.7±5.8	31.5±19.6	13.1±16.5^b^	Not reported
Sariyildiz (19)	70	60	36.4±7.4	35.2±7.7	3.1±2.0	2.3±1.9	9.9±6.9	28.2±33.3	18.7±13.7	4.5±5.5	6.7±5.2
Demir (20)	23	27	39.34±6.28	37.58±9.58	2.16±2.05	4.02±2.18	8.6±5.0	Not reported	24.04±19.19^c^	1.48±4.16^c^	7.33±4.26
Sariyildiz (21)	37	33	34.1±7.0	33.5±6.2	3.9±2.3	4.5±1.9	8.6±7.4	32.9±32.4	16.5±8.9	4.2±6.3	6.8±3.1

Values presented are mean ± standard deviation. BASFI, Bath AS functional index; BASDAI, Bath AS disease activity index; DD, disease duration; DMS, duration of morning stiffness; ESR, erythrocyte sedimentation rate; CRP, C-reactive protein; ASQoL, ankylosing spondylitis quality of life questionnaire; AS, ankylosing spondylitis.

a Without standard deviation;

b in units of g/dl;

c without units.

**Table II:** Results of meta-analysis for male sexual function.

	Heterogeneity			Test for overall effect		
		
Outcomes	χ^2^	P-value	I^2^ (%)	Z	P-value	MD (95% CI)
EF	2.21	0.33	9	5.57	<0.00001	−3.07 (−4.15, −1.99)
IS	4.38	0.11	54	6.93	<0.00001	−1.89 (−2.42, −1.35)
OF	6.30	0.04	68	2.73	0.006	−1.17 (−2.00, −0.33)
SD	3.18	0.20	37	4.32	0.0001	−0.72 (−1.05, −0.40)
OS	14.88	0.0006	87	1.73	0.08	−0.94 (−2.01, 0.12)

MD, mean difference; CI, confidence interval; EF, erectile function; IS, intercourse satisfaction; OF, orgasmic function; SD, sexual drive; OS, overall satisfaction.

**Table III:** Results of meta-analysis for female sexual function.

	Heterogeneity			Test for overall effect		
		
Outcomes	χ^2^	P-value	I^2^ (%)	Z	P-value	MD (95% CI)
Desire	0.33	0.57	0	2.15	0.03	−0.34 (−0.65, −0.03)
Arousal	1.74	0.19	43	4.61	<0.00001	−0.87 (−1.24, −0.50)
Lubrication	8.43	0.004	88	0.74	0.46	−0.41 (−1.51, 0.68)
Orgasm	9.24	0.002	89	0.59	0.55	−0.37 (−1.60, 0.86)
Satisfaction	6.92	0.009	86	0.28	0.78	−0.15 (−1.19, 0.90)
Pain	1.33	0.25	25	0.90	0.37	−0.20 (−0.63, 0.23)
Total FSFI	7.15	0.007	86	0.80	0.42	−2.08 (−7.17, 3.01)

MD, mean difference; CI, confidence interval; FSFI, female sexual function index.
